# Influence of Oregano Essential Oil on the Rumen Microbiome of Organically Reared Alpine Goats: Implications for Methanobacteria Abundance

**DOI:** 10.3390/ani15131937

**Published:** 2025-07-01

**Authors:** Dimitrios Kyrtsoudis, Maria V. Alvanou, Dimitrios Loukovitis, Dimitrios Gourdouvelis, Vasileios A. Bampidis, Dimitrios Chatziplis, Ioannis K. Mitsopoulos

**Affiliations:** 1 Department of Agriculture, School of Geosciences, International Hellenic University, 57400 Sindos, Greece; bampidis@ihu.gr (V.A.B.); chatz@ihu.gr (D.C.); gmitsop@ihu.gr (I.K.M.); 2 Faculty of Agriculture, Forestry and Natural Environment, Aristotle University of Thessaloniki, 54621 Thessaloniki, Greece; malvano@agro.auth.gr; 3 Laboratory of Applied Genetics, Department of Fisheries & Aquaculture, School of Agricultural Sciences, University of Patras, 30200 Mesolonghi, Greece; 4 Animal Genetic Improvement Institute of Thessaloniki, Nea Mesimvria, 57011 Thessaloniki, Greece; topidaias@gmail.com

**Keywords:** dairy goats, Alpine, *Origanum vulgare*, methanobacteria, archaea

## Abstract

The present study aimed to evaluate the effects of dietary supplementation with organic oregano (*Origanum vulgare*) essential oil (OEO) on the rumen microbial population, with a focus on methanogenic archaea, in lactating dairy goats. A total of nine age-matched goats (mean body weight 49 ± 1.8 kg) were assigned to three experimental groups (*n* = 3 per group) in a completely randomized design. All animals were fed a basal diet consisting of a corn-based concentrate and a forage mix composed of alfalfa hay, wheat straw and corn silage. Group 1 was the control group while Groups 2 and 3 received an OEO supplement at dosages of 1 mL/day and 2 mL/day per animal, respectively, incorporated into the concentrate feed. Rumen fluid samples were collected on days 15, 30 and 45 of the feeding trial and their microbial profile was assessed using NGS analysis. The results demonstrated a reduction in the relative abundance of methanobacteria in both OEO-supplemented groups compared to the control group. Statistical analysis revealed significant differences between feeding groups and days of sampling. These findings suggest that OEO has the potential to modulate the rumen microbiome by reducing methane-producing archaeal populations. In conclusion, dietary supplementation with OEO may serve as a natural strategy to mitigate enteric methane emissions in Alpine dairy goats.

## Simple Summary

The reduction of methane emissions originating from the livestock sector is of high importance towards the confrontation of climate change. In the present study, the effect of organic oregano (*Origanum vulgare*) essential oil (OEO) incorporated into the feeding ratio in lactating dairy goats over a 45-day period, on the abundance of the Methanobacteria population in their rumen was investigated. Samples were collected on days 15, 30 and 45 of the feeding trial to investigate the alterations in microbial populations, using both generic microbial and archaeal-specific primer pairs. From the results, it occurred that the animals receiving OEO were characterized by lower Methanobacteria abundances over time compared to animals that did not receive the supplementation with OEO. This effect was observed using both analytical approaches targeting generic and archaeal-specific microbial communities. These findings are important because they show a promising, environmentally friendly method for making livestock farming more sustainable.

## 1. Introduction

Methane (CH_4_) is a potent greenhouse gas with a global warming potential approximately 28 times greater than that of carbon dioxide (CO_2_) over a 100-year timescale and up to 80 times more potent over 10–20 years [1]. Agriculture is a major anthropogenic source of CH_4_, primarily through enteric fermentation in ruminants, manure management, rice paddy cultivation and crop residue burning [2]. Among these, enteric fermentation alone contributes to approximately 30% of total atmospheric methane emissions and represents the largest single source within livestock production systems, accounting for roughly 40% of greenhouse gas (GHG) emissions from the livestock sector and 6% of total global anthropogenic GHG emissions [3,4,5,6].

According to FAOSTAT, livestock-related emissions are not only substantial but also increasing, especially in regions with growing demand for animal-source foods [2]. This trend exacerbates concerns surrounding climate change, as global average temperatures have increased by more than 1.2 °C since the 1960s, threatening sensitive ecosystems and accelerating biodiversity loss [7].

Ruminant animal production is dependent on the anaerobic microbial ecosystem (including bacteria, archaea, protozoa and fungi) to ferment and transform human indigestible forages into high-grade dairy and meat products for human consumption. Ruminant animals, however, are major emitters of enteric methane (CH_4_) due to the microbial breakdown of carbohydrates in the rumen [8,9], representing an unproductive loss of dietary energy [10]. This symbiotic process enables ruminants to convert low-quality forages into nutrient-dense meat and milk, essential for global food security [11]. Enteric CH_4_ is produced under anaerobic conditions by a diverse community of methanogenic archaea, using mainly hydrogen and CO_2_ as substrates [12]. The quantity of feed consumed by a ruminant largely determines the quantity of CH_4_ emitted, though the type and quality of the animal feed also influence emissions [13,14]. The species of the ruminant, the individual’s digestive physiology and the makeup of the resident microbial population can also influence the quantity of the CH_4_ it produces [15,16,17].

However, methanogenesis, being the final step in the fermentation process, involves archaea that utilize hydrogen and carbon dioxide to produce CH_4_, representing an energy loss of approximately 6–12% of gross energy intake (GEI) or 8–14% of digestible energy intake (DEI) [6,8]. This inefficiency imposes both environmental and economic costs [10,18,19].

Factors influencing enteric CH_4_ emissions include feed intake level, diet composition, the ruminant species, digestive physiology, and the structure and function of the rumen microbiome [9,10,12,13]. Consequently, targeted mitigation strategies, such as dietary manipulation, feed additives, breeding programs and microbiome engineering, are increasingly under investigation to reduce CH_4_ emissions while improving productivity [14].

One promising strategy involves the supplementation of ruminant diets with plant-derived essential oils, such as oregano essential oil (OEO), which has shown antimicrobial properties against specific groups of rumen microbes, particularly methanogenic archaea. Research indicates that OEO can significantly reduce CH_4_ production by inhibiting the activity and abundance of Methanobacteriales, a dominant group of archaea responsible for hydrogenotrophic methanogenesis. Additionally, OEO alters the broader microbial ecosystem, potentially enhancing propionate production, which serves as a hydrogen sink and reduces substrate availability for methanogenesis [15]. While effects may vary depending on dose and diet composition, the use of OEO represents a promising natural additive to mitigate CH_4_ emissions sustainably. Carvacrol, the main component of OEO used in this study (92.80%), is known to exhibit antimicrobial activity affecting the lipid membrane of methanogen bacteria, potentially disrupting methanogenesis [20,21,22].

Thus, the scope of the present study was to investigate the impact of supplementing the diet of lactating dairy goats with organic OEO on rumen microbial communities, with an emphasis on methanogenic archaea.

## 2. Materials and Methods

### 2.1. Goats, Experimental Design and Diets

In order to carry out the experiment, a random sample of nine milk-producing goats of Alpine breed were selected from a total herd of 450 goats kept in the Regional Unit of Evros, Greece. Selection criteria were only the same age and lactating period of the selected goats (age 4 years ± 1 months, 5th parity), which had an average body weight of 49 ± 1.8 kg.

The above nine goats were divided into three treatment groups, each consisting of three animals (triplicate). Group 1 was the control and was given the standard binary ration of roughage and concentrated organic feed. In groups 2 and 3, 1 mL and 2 mL of organic OEO were added, respectively, to the concentrate feed mixture per animal per day. The ration was provided twice—in the morning and in the afternoon—after milking.

The daily roughage supply reached 2.3 kg and consisted of 1.3 kg alfalfa hay, 0.3 kg wheat straw and 0.7 kg maize silage. Regarding the concentrated feed mixture, the daily supply reached 1.2 kg and its composition was 50% corn, 20% soybean meal, 13% bran, 6% barley, 6% triticale and 5% inorganic and vitamin premix. The incorporation of organic OEO into the ration was performed using Tween 80 (Polysorbate 80) emulsifier, in a quantity of 2 mL per animal per day in each group, including the control. During the experiment, the goats were kept in group pens of three individuals and had free access to clean water. The OEO analysis is depicted in Table 1.

Polysorbates are widely utilized surfactants in biopharmaceutical formulations for their role in mitigating protein denaturation, aggregation and surface adsorption. Polysorbate 80 can be produced by the esterification, polyetherification and reesterification of sorbitol with plant-derived oleic acid [23]. The daily mixing and feeding protocols keep any possible degradation of Polysorbate 80 to a minimum. The inclusion of Polysorbate 80 in the basal diet (in all 3 groups), mitigated potential biases, if any, between all 3 separate treatments.

After a 15-day adjustment period, where all animals were fed the standard binary ration, three different dietary treatments started on the 25th day of lactation and lasted for 45 days (last day of the experiment was the 70th day of lactation). Rumen liquid samples were taken from each animal using a gastroesophageal sampling tube, on the 15th, 30th and 45th day of the trial (nine samples from each time point, 27 samples in total). All liquid samples were immediately stored in −80 °C for the subsequent isolation of microbial DNA. A minimum of 60 mL of sample was collected from each animal using a plunger along with the gastroesophageal sampling tube. While being a standard procedure, to combat potential biases (e.g., cross-contamination between animals), all sampling tubes and plungers were of medical grade, sterilized before use and discarded after each individual sampling.

### 2.2. DNA Extraction, Sequencing and Taxonomy Analysis

Microbial genomic DNA was extracted from each one of the 27 collected liquid samples, using 1 mL of liquid as starting material, with the DNeasy PowerSoil Pro Kit (Qiagen, Hilden, Germany). A spectrophotometry method (Q3000 microvolume spectrophotometer, Quawell, San Jose, CA, USA) was applied to check the quantity and quality of DNA samples.

For microbial diversity evaluation, two primer pairs were used targeting the V3-V4 hypervariable region of the *16S rRNA* gene. The first one, Pro341F (5′-CCTACGGGNBGCASCAG-3′) and Pro805R (5′-GACTACNVGGGTATCTAATCC-3′), is a universal prokaryotic primer pair for the simultaneous amplification of bacteria and archaea, while the second one, ARC344F (5′-ACGGGGYGCAGCAGGCGCGA-3′) and Arch806R (5′-GGACTACVSGGGTATCTAAT-3′), is specific to Archaea amplification [24,25,26]. PCR amplification was conducted with the KAPA2G™ Robust HotStart ReadyMix PCR Kit (Kapa Biosystems, Wilmington, MA, USA) in a total volume of 15 μL, along with PCR-grade water, the primer pair (50 nM each) and DNA (~50–100 ng). The following touchdown PCR protocol (same for both primer sets) was employed: initial denaturation at 98 °C for 2 min, followed by 35 cycles at 98 °C for 30 s, annealing starting from 65 °C and ending at 55 °C for 15 s, 68 °C for 30 s, and a final elongation step at 68 °C for 5 min. The annealing temperature was decreased by 1 °C in each cycle until reaching 55 °C, which was kept for the remaining 25 cycles. In order to confirm the successful amplification of the *16S rRNA* amplicons, these were electrophorized in agarose gel, while their purity and concentration were examined again with spectrophotometry.

The obtained amplicons were subsequently purified with AMPure^®®^ XP beads (Beckman Coulter, Brea, CA, USA) and prepared for sequencing through ligation, using the Native Barcoding Kit 96 V14 (SQK-NBD114.96, Oxford Nanopore Technologies plc., Oxford Science Park, Oxford, UK). The constructed DNA library containing a pool of 54 barcoded PCR products (27 from each primer pair), was run in a MinION-Mk1B sequencing device with a R.10.4.1 flowcell (Oxford Nanopore Technologies plc., Oxford Science Park, Oxford, UK).

The demultiplexing (according to the used barcodes) and basecalling of raw sequencing reads was done with Dorado software version 1.0.2 (Oxford Nanopore Technologies plc., Science Park, Oxford, UK), where barcodes, adapter and primer sequences were also removed in order to obtain clean *16S rRNA* sequences. In addition, only those reads with a length between 350–550 bp and a quality score (Q-score) above 15 were kept.

For taxonomy analysis, QIIME 2 2024.5.0. statistical package [27] and the GSR database were used (https://manichanh.vhir.org/gsrdb/) accessed on 15 March 2025 [28], setting 95% as a minimum percentage of identity for successful classification. For each primer set, the 27 sequenced PCR products were grouped (one group per triplicate) based on dietary treatment (groups 1, 2 and 3) and sampling time-point (days 15, 30 and 45) information, concluding in nine sample groups in total (Group 1_15, Group 2_15, Group 3_15, Group 1_30, Group 2_30, Group 3_30, Group 1_45, Group 2_45 and Group 3_45). Taxa barplots were created in order to examine the degree of microbial diversity, based on the composition and abundance of bacterial and/or archaeal communities in each sample group. Alpha diversity indices, i.e., the Shannon diversity index and Simpson’s diversity index [29,30], have also been calculated for each sample group using QIIME 2 version 2024.5.0 software. Regarding beta diversity analysis, Emperor plots were constructed based on the Bray–Curtis dissimilarity index [31]. Finally, an alpha rarefaction analysis was employed at the genus level in order to examine the adequacy of sequencing depth for each barcoded PCR sample.

### 2.3. Statistical Analysis

The data of the analyzed samples related to the microbe populations (percentages) of the rumen were considered as the variables, and the date of the sampling and the mean milk yield of each group were considered as the variables entered into the SPSS for statistical processing. A Generalized Linear Model (GLM-Multivariate Analysis) was used with the percentage of Methanobacteria being the dependent variable, milk yield as a covariate and the group-treatment and day of sampling (experiment day) as fixed factors. All factors and covariates were examined for the main effects and groups, while days were additionally examined pairwise for significant differences. The same procedure was followed for both primer sets (universal prokaryotic and archaeal). All statistical analyses were carried out using the statistical package SPSS 30.

## 3. Results

### 3.1. Microbial Diversity Analysis

Regarding the universal prokaryotic primer pair (Pro341F/Pro805R), 913.744 reads were obtained after the basecalling and filtering steps of the raw sequencing data. The number *16S rRNA* amplicon reads per sample group ranged from 69.540 to 126.664, with an average of 101.527 reads. For the archaeal primer set (ARC344F/Arch806R), 1.031.061 clean reads were obtained ranging from 86.079 to 158.699 per sample group, having a mean of 114.562 reads.

**Figure 1:**
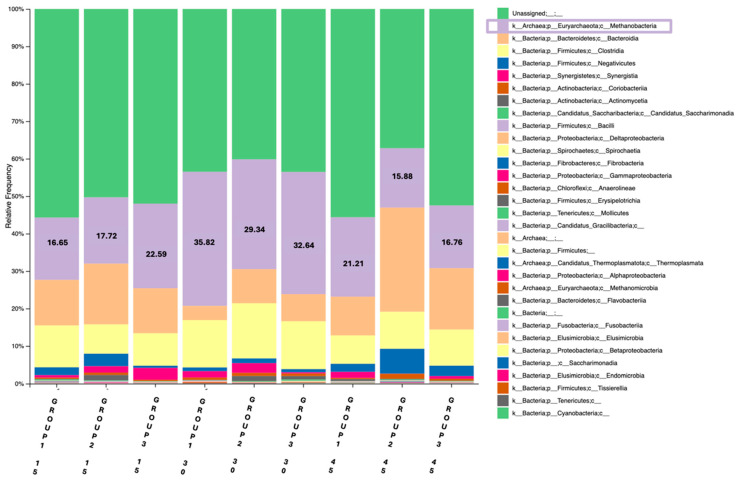
Relative abundance of Methanobacteria (%) in the rumen of goats across the three groups (Group 1: Control, Group 2: 1 mL OEO/day and Group 3: 2 mL OEO/day), in the three different time-points of the feeding trial (days 15, 30 and 45), using the universal prokaryotic primer pair.

**Figure 2:**
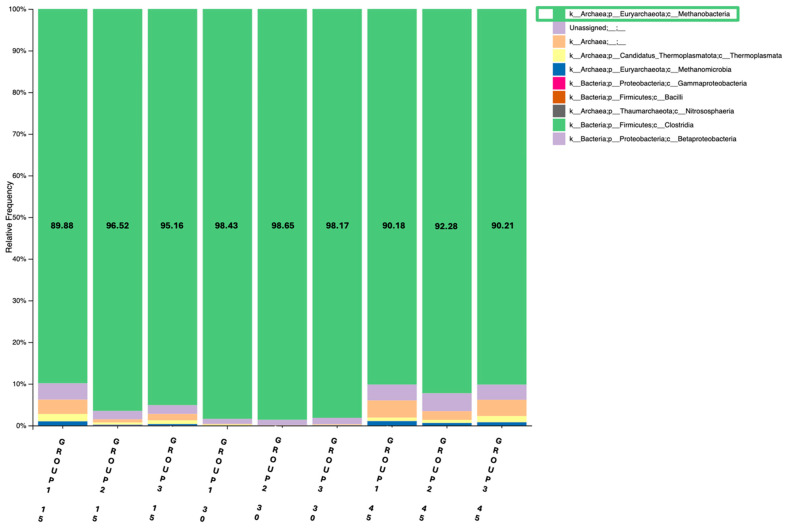
Relative abundance of Methanobacteria (%) in the rumen of goats across the three groups (Group 1: Control, Group 2: 1 mL OEO/day and Group 3: 2 mL OEO/day), in the three different time-points of the feeding trial (days 15, 30 and 45), using the archaeal primer set.

One barplot was constructed using data obtained from the universal prokaryotic primer pair (Pro341F/Pro805R), providing a broader overview of the prokaryotic community (Figure 1). The second barplot was based on the archaeal primer set (ARC344F/Arch806R), that enables a focused assessment of methanogenic archaea (Figure 2). Regarding the first barplot on day 15, the control group (Group 1) exhibited a Methanobacteria abundance of 16.65%, while Group 2 and Group 3 showed 17.72% and 22.59%, respectively. By day 30, the methanobacteria abundance was increased in all three groups, reaching 35.82%, 29.34% and 32.64%, respectively. On day 45, a notable decrease was observed across all groups; Methanobacteria abundance declined to 21.21% in Group 1, 15.88% in Group 2 and 16.76% in Group 3. However, when comparing the abundance results between sampling days 15 and 45, groups 2 and 3 displayed a decrease and Group 1, on the other hand, presented an increase in Methanobacteria abundance. This visual representation demonstrates a consistent pattern across all groups, with Methanobacteria abundance reaching its highest levels during peak lactation (day 30), followed by a marked decrease in the later phase.

The second barplot revealed a similar pattern regarding the abundance of methanobacteria between the different sampling days. More specifically, Methanobacteria were increased in all feeding groups from day 15 to day 30, followed by a reduction on day 45 (Figure 2). On day 30, the percentage of Methanobacteria was approximately 98% in all three groups, aligning with the increased energy demands during the lactation peak, followed by a reduction as energy requirements declined. Again, Group 1 showed a slight increase in Methanobacteria between 15 and 45 time-points, whereas groups 2 and 3 presented a decrease of abundance in the above time frame.

Finally, the obtained results from alpha (Shannon and Simpson’s indices) and beta (Emperor plots) diversity analysis, as well as the generated rarefaction curves are provided as Supplementary Material.

### 3.2. Microbial Abundance and Milk Yield Correlation

**Figure 3:**
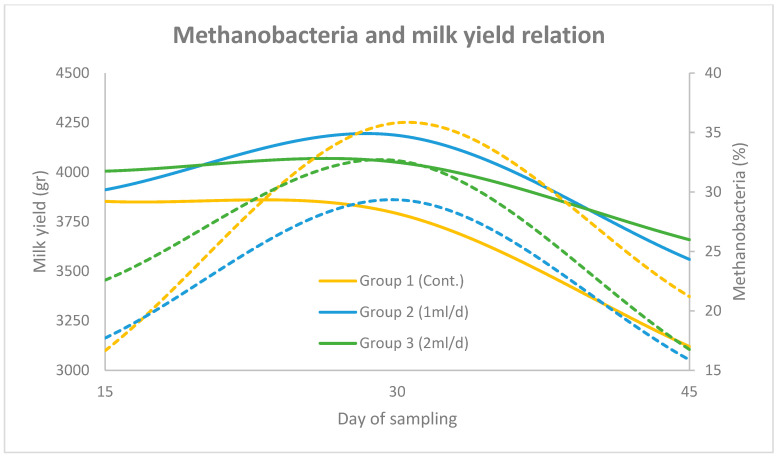
Relationship between Methanobacteria (dashed lines) and milk yield (solid lines) across three sampling points (days 15, 30 and 45) for Groups 1, 2 and 3, using the universal prokaryotic primer pair. Methanobacteria percentages are plotted on the right y-axis and milk yield (g) on the left y-axis.

**Figure 4:**
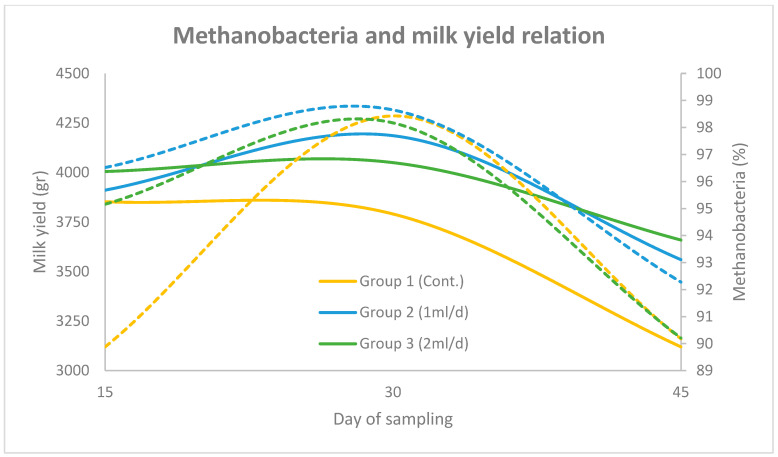
Relationship between Methanobacteria (dashed lines) and milk yield (solid lines) across three sampling points (days 15, 30 and 45) for Groups 1, 2 and 3, using the archaeal primer set. Methanobacteria percentages are plotted on the right y-axis and milk yield (g) on the left y-axis.

In Figure 3 and Figure 4 the correlation between Methanobacteria abundance and milk yield (group mean) over the three sampling periods (days 15, 30 and 45), across the three experimental groups (using the two primer sets), is illustrated. The solid lines represent the progression of milk yield, while the dashed lines denote Methanobacteria proportions. The results obtained using the universal prokaryotic and the archaeal primer pairs follow the same motif. More specifically, in Group 1, milk yield increased steadily to a peak on day 30 before declining, which coincided with a sharp rise in Methanobacteria levels. In Group 2, milk yield followed a similar trend, though Methanobacteria levels peaked slightly earlier and remained lower than Group 1 throughout the trial. In Group 3, both milk yield and Methanobacteria levels were more stable, with a moderate peak on day 30 and a noticeable reduction in Methanobacteria abundance by day 45.

Moreover, GLM-Multivariate analysis revealed that Methanobacteria abundance was significantly influenced by the day of sampling, treatment group and overall group milk yield. For both primer sets, a strong time-dependent effect was observed indicating that methanogenic bacterial populations changed over the course of the lactation period. Additionally, goats supplemented with OEO showed a statistically significant reduction in methanogen abundance compared to Group 1, supporting the hypothesis that OEO exerts a suppressive effect on methane-producing microbes. Furthermore, milk yield was also significantly associated with methanogen abundance, suggesting a link between productive performance and rumen microbial dynamics (Table 2). Regarding the variable of milk yield, the results showed a significant correlation to the microbe population composition, with the groups with the OEO inclusion presenting a greater milk yield and smaller methanogen populations, especially on day 45. Further, group and day of sampling variables also had a significant effect on the microbe population, with Groups 2 and 3 presenting significantly lesser methanogen archaea populations on day 45 (Table 2).

Pairwise comparisons between sampling days further confirmed the temporal shifts in Methanobacteria abundance across the lactation period. Significant differences were observed between all time points, including between day 15 and day 30, day 30 and day 45, and day 15 and day 45 for both pairs of primers. These findings indicate that the rumen methanogen population responded dynamically over time, with notable changes occurring throughout the course of OEO supplementation and lactation (Table 3).

Additionally, pairwise comparison analysis for the group-treatment, regarding the universal prokaryotic primer pair, revealed significant differences between Group 1 and Group 3, as well as, between Group 2 and Group 3. However, no significant difference was found between Group 1 and Group 2. These results suggest a dose-dependent effect of OEO supplementation on reducing methanogenic archaea, with Group 3 showing the most pronounced impact. On the other hand, the pairwise comparison analysis for the group-treatment using the archaeal primer pair revealed significant differences between Group 1 and Group 2, as well as between Group 1 and Group 3. However, no significant difference was found between Group 2 and Group 3. These findings suggest an effect of OEO supplementation on reducing methanogenic archaea within the archaea populations (Table 4).

For a better interpretation of the results regarding the long-term effect of OEO on methanobacteria abundance, a statistical comparison was performed between feeding groups, omitting the data from day 30. The analysis revealed differences across all pairwise comparisons: Group 1 versus Group 2, Group 1 versus Group 3, and Group 2 versus Group 3, being statistically significant for both pairs of primers. Since all P-values were well below 0.05, the null hypothesis of no group differences can be confidently rejected (Table 5). These findings support the conclusion that OEO supplementation had a measurable impact on the abundance of Methanobacteria. Interestingly, an increase in Methanobacteria levels was observed across all groups on day 30, which coincided with the peak of lactation and elevated milk production.

## 4. Discussion

In the present study, the impact of dietary supplementation with OEO on the rumen microbial composition of lactating dairy goats, with an emphasis on methanogenic archaea, was investigated. From the controlled feeding trial that was conducted, the three feeding groups were differentiated by the inclusion of 0, 1, or 2 mL of OEO per goat per day in the concentrate. Rumen fluid was sampled at three time points (days 15, 30 and 45 of the feeding trial) to assess changes in microbial populations. The primary aim was to evaluate whether organic OEO could reduce the abundance of methane-producing microorganisms, thereby offering a potential strategy for lowering enteric methane emissions in small ruminant production systems. Overall, the administration of organic OEO in Alpine goats decreased methane-producing microbe populations compared to the control group. More specifically, a consistent pattern emerged across both primer sets (universal prokaryotic and archaeal ones) indicating a notable reduction in methanogenic archaea from day 15 to day 45 in both feeding groups (Group 2 and Group 3), while the control group (Group 1) showed an increasing trend over the same period. This trend supports the hypothesis that OEO supplementation exerts a suppressive effect on methanogen populations within the rumen. Interestingly, the decline in Methanobacteria abundance was more pronounced in Group 3 than in Group 2, particularly evident at day 45, suggesting that the 2 mL/day dosage may have exerted a more targeted antimicrobial effect against methanogens than the 1 mL/day dose. The similar trends obtained from both the bacterial and archaeal sequencing data strengthen the validity of the observed reductions, underscoring the consistency of the OEO effect across the different analytical approaches.

The temporal evaluation of Methanobacteria abundance revealed a time-dependent response to OEO supplementation. Even though differences between groups were modest at day 15, a more discernible divergence emerged by day 45, indicating a delayed but clearer suppressive effect of OEO on methanogenic populations. Notably, on day 30 of the feeding trial, coinciding with the peak of the lactation (day 55), the Methanobacteria abundance remained relatively elevated across all groups, including those receiving OEO. This suggests that during periods of high-energy demand, such as active milk production, ruminants may prioritize energy-yielding fermentation pathways, potentially mitigating the suppressive impact of OEO on methanogenesis. The clearer reduction in Methanobacteria by day 45 implies that as lactation energy demands diminish, the ruminal microbial community may become more responsive to the modulatory effects of OEO. This observation underscores the dynamic interplay between host physiological status and microbial ecology, suggesting that the efficacy of dietary interventions aimed at reducing methane emissions may vary across different production stages.

From a previous study involving Chios sheep, the administration of ropadiar, an oregano-derived essential oil, at a dosage of 250 mg/day resulted in lower methane output than in control animals [32]. Moreover, Chios dairy ewes fed a concentrate diet supplemented with 100 or 150 mg/kg of a blended EO showed reductions in rumen urea, a lower acetate-to-propionate ratio, a rise in cellulolytic bacterial populations, and fewer hyper-ammonia-producing microbes [33]. Furthermore, essential oils such as those from clove, eucalyptus, garlic, oregano and peppermint have been observed to reduce populations of archaea, protozoa and cellulolytic bacteria in the rumen of lactating cattle [34], which may explain the observed decrease not only in methane production but also in fiber digestion.

In line with the above results, the present study demonstrated that the inclusion of lower doses of organic OEO (1 mL and 2 mL per animal per day) in the diets of lactating dairy goats influenced the rumen microbial population, particularly methanogenic archaea, in a direction that may support reduced methane emissions. While differences in dosage and animal species should be taken into account, the consistency in microbial shifts across studies supports the hypothesis that moderate EO inclusion can modulate rumen fermentation dynamics as it affects methanogenic archaeal populations. Nonetheless, the limited number of experimental animals and the complex nature of EO bioactivity highlight the necessity for more extensive trials to unravel the mechanisms underpinning these effects.

Furthermore, Paraskevakis [35] examined the long-term administration of dried *Origanum vulgare* ssp. hirtum in non-lactating Alpine goats. A significant reduction in the total methanogen population was demonstrated, alongside an enhanced protease activity and elevated ammonia concentrations in the rumen. While both studies employed oregano-based interventions to suppress methanogenesis, differences in form (dried whole plant versus extracted EO), dosage strategy and production stage of the animals (non-lactating vs. lactating) likely influenced the magnitude and kinetics of the observed microbial shifts. In our study, the reduction in methanogen abundance became more apparent by day 45 of supplementation. These results suggest that liquid OEO may exert a comparable if not more targeted antimicrobial effect against methanogens than dried OPs, potentially due to higher bioavailability and standardized concentrations of active compounds such as carvacrol and thymol. Carvacrol, a major bioactive phenolic compound in OEO, has been shown to exert antimicrobial effects by disrupting the cell membrane integrity of various microorganisms by integrating into the lipid bilayer, altering membrane permeability and leading to the leakage of cellular contents and disruption of ion gradients [20,21]. In methanogens, such disruption compromises membrane-bound enzymes essential for methanogenesis, such as methyl-coenzyme M reductase (MCR), which catalyzes the final step in methane production [22].

Research conducted on other species, more specifically rabbits, indicated that the microbial effect of OEO not only enhanced growth performance, feed efficiency and meat quality, but also lower cholesterol levels and boosted antioxidant activity [36]. Those results not only strengthen the use of OEO as an alternative to synthetic antimicrobials, but also its beneficial effect on carcass quality could improve the production and cost effectiveness on farms following a mixed production path, if the same results can apply for small ruminants.

However, the results of the present study come in contrast with the findings reported by Benchaar et al. [37], who observed no significant effects of OEO or its main active compound, carvacrol, on enteric methane (CH_4_) production, nutrient utilization, nitrogen excretion or milk performance in dairy cows supplemented at 50 mg/kg of dietary dry matter. Other in vivo studies on the supplementation of oregano-based products in ruminant diets have also presented contrasting results regarding their impact on enteric methane emissions and rumen microbial dynamics. For instance, Lejonklev et al. [38] reported no detectable effects on dry matter intake (DMI), milk yield, or enteric CH_4_ emissions following the high-dose short-term administration of oregano oil, while Tekippe et al. [39] and Hristov et al. [40] demonstrated modest reductions in CH_4_ production after pulse dosing oregano leaves in dairy cows. However, these reductions were not accompanied by significant changes in rumen fermentation characteristics, nutrient digestibility, or protozoa abundance. The discrepancy between the studies may stem from species-specific digestive physiology and microbial ecosystem dynamics, as goats and cows exhibit differences in their rumen microbiota structure and fermentation patterns. More specifically, Khiaosa-Ard and Zebeli [41] reported that EOs had a more pronounced effect in lowering acetate concentrations and methane emissions in small ruminants compared to dairy cows. Additionally, the feeding method and concentration of OEO could play critical roles in modulating its efficacy. The use of higher absolute doses in our study (in mL per animal) and the potential synergistic effects of OEO’s multiple constituents, beyond carvacrol alone, may have contributed to the antimicrobial effect on methanogenic populations observed. These findings underscore the importance of dose, administration method, and species-specific responses when evaluating the efficacy of phytogenic feed additives in ruminant nutrition.

To conclude, the present study highlights organic OEO’s potential as a natural feed additive capable of modulating the rumen microbial environment in Alpine dairy goats during lactation periods by reducing the population of methanogenic archaea. The observed time-related pattern, where changes in methanobacteria abundance became more prominent at 45 days in comparison to 30 days, underscores the influence of physiological stage on the effectiveness of such dietary interventions. Overall, our findings support the idea that these bioactive compounds can contribute to methane mitigation strategies in small ruminants. Nonetheless, the complexity of rumen microbial networks and the variation in animal responses call for further investigation to refine dosing strategies and better understand the long-term implications on animal health of essential oil supplementation in ruminant nutrition.

## 5. Conclusions

In the present study the effects of organic OEO supplementation on the rumen microbial ecosystem of lactating Alpine dairy goats were investigated using two different analytical approaches. Based on the results, a gradual reduction in methanogen abundance was revealed, related to dosage, becoming more evident by day 45 of the feeding trial, suggesting that the suppressive effect of OEO becomes more pronounced as lactational energy demands decline. Our findings align with previous research showing that oregano-derived compounds can modulate ruminal fermentation and reduce methane emissions, although responses may vary depending on species, physiological stage, dosage and integration method. The results herein support the hypothesis that goats may be more responsive to the antimicrobial properties of OEO, possibly due to differences in the rumen physiology of small ruminants. Furthermore, the liquid form of OEO may offer improved bioavailability and more consistent active compound concentrations compared to dried plant material. Overall, the inclusion of OEO in the diet of lactating goats revealed promising results operating as a natural strategy for mitigating enteric methane emissions. Further investigation of the underlying mechanism on the dependence of the rumen microbiota on OEO supplementation, the effects of OEO on small ruminant productive traits (e.g., carcass quality and weight, milk yield, lipid profile, protein-fat-lactose content etc.) as well as the correlation of such traits with methanogen archaea populations, is deemed necessary. Using larger sampling sizes (more animals), more treatment groups, as well as more in-depth molecular genetic analysis (e.g., a full gene sequencing) and a longer experimental time with more sampling time-points, are some key methods of obtaining more statistical data, yielding more robust results.

## Figures and Tables

**Table 1:** Chemical composition of the organic oregano essential oil.

Compound	Yield (%)
α-thujene	0.04
α-pinene	0.06
1-octen-3-ol	0.03
β-myrcene	0.09
α-terpinene	0.13
p-cymene	0.73
β-phellandrene	0.07
γ-terpinene	0.52
α-terpinolene	0.04
borneol	0.33
terpinen-4-ol	0.40
α-terpineol	0.14
carvacrol methyl ether	0.05
thymol	2.22
carvacrol	92.80
β-caryophyllene	0.99
α-humulene	0.13
β-bisabolene	0.72
δ-cadinene	0.07
caryophyllene oxide	0.24

**Table 2:** Effect of sampling day, treatment group and milk yield on the abundance of Methanobacteria in the rumen of lactating goats, using the universal prokaryotic and archaeal primer pairs.

	Universal Prokaryotic Primer Pair	Archaeal Primer Pair
Variable	*p*-Value	*p*-Value
Day of sampling	0.000 *	0.000 *
Group	0.002 *	0.000 *
Milk yield	0.000 *	0.000 *

* statistically significant (*p* < 0.05) after the Bonferroni correction.

**Table 3:** Pairwise comparisons of Methanobacteria abundance across sampling days in lactating Alpine goats, using the universal prokaryotic and archaeal primer pairs.

	Universal Prokaryotic Primer Pair	Archaeal Primer Pair
Comparison Between Days	*p*-Value	*p*-Value
Day 15 and 30	0.000 *	0.000 *
Day 30 and 45	0.000 *	0.000 *
Day 15 and 45	0.000 *	0.000 *

* statistically significant (*p* < 0.05) after the Bonferroni correction.

**Table 4:** Pairwise comparison of Methanobacteria abundance between treatment groups using the universal prokaryotic and archaeal primer pairs.

	Universal Prokaryotic Primer Pair	Archaeal Primer Pair
Comparison Between Groups	*p*-Value	*p*-Value
Group 1 and 2	0.391	0.000 *
Group 2 and 3	0.010 *	0.087
Group 1 and 3	0.002 *	0.000 *

* statistically significant (*p* < 0.05) after the Bonferroni correction.

**Table 5:** Pairwise statistical comparison of Methanobacteria abundance between feeding groups (excluding day 30), using the universal prokaryotic and archaeal primer pairs.

	Universal Prokaryotic Primer Pair	Archaeal Primer Pair
Comparison Between Groups	*p*-Value	*p*-Value
Group 1 and 2	0.005 *	0.000 *
Group 2 and 3	0.000 *	0.001 *
Group 1 and 3	0.000 *	0.000 *

* statistically significant (*p* < 0.05) after the Bonferroni correction

## Data Availability

The raw data supporting the conclusions of this article will be made available by the authors on request.

## Supplementary Materials

The following supporting information can be downloaded at: https://www.mdpi.com/article/10.3390/ani15131937/s1, Figure S1: Rarefaction curves of all barcoded 16s rRNA amplicon samples at the genus level, using: (a) the archaeal primer set (ARC344F/Arch806R) and (b) the universal prokaryotic primer pair (Pro341F/Pro805R); Figure S2. 3D Emperor plot for the nine sample groups under study, based on the Bray–Curtis dissimilarity index at the genus level, using: (a) the archaeal primer set (ARC344F/Arch806R) and (b) the universal prokaryotic primer pair (Pro341F/Pro805R); Table S1. Shannon diversity index and Simpson’s diversity index for the nine sample groups under study, using the archaeal primer set (ARC344F/Arch806R) and the universal prokaryotic primer pair (Pro341F/Pro805R).
