# Supplementary material for: Influence of Oregano Essential Oil on the Rumen Microbiome of Organically Reared Alpine Goats: Implications for Methanobacteria Abundance
Source: Animals (Basel). 2025 Jul 1;15(13):1937. doi: 10.3390/ani15131937 (PMC12249385; doi:10.3390/ani15131937)
Supplement: Supplementary file 1 [file animals-15-01937-s001.zip › animals-3682745-supplementary.pdf]

(a)

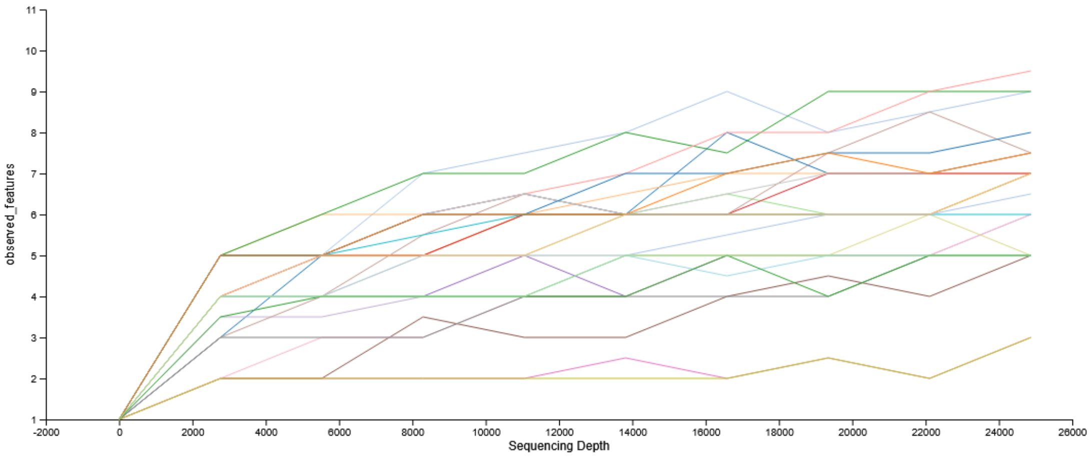

(b)

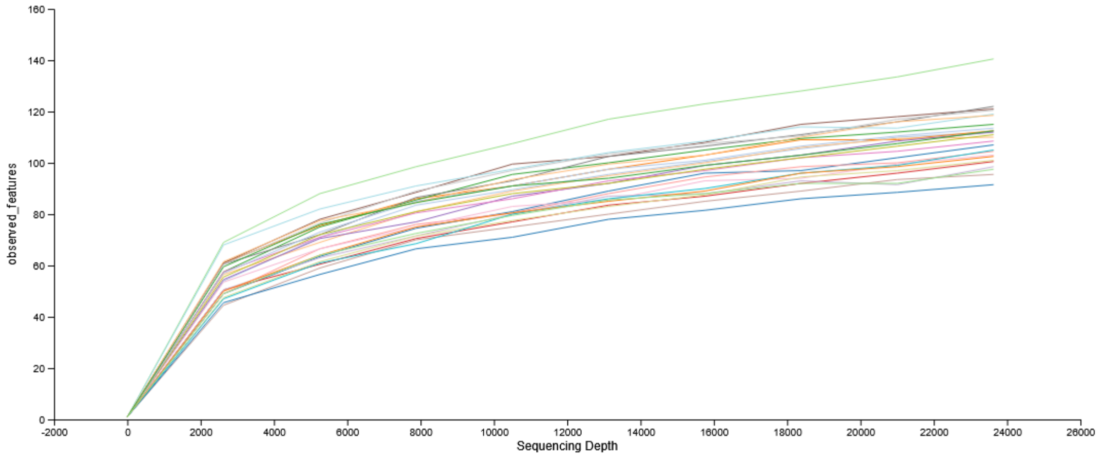

Figure S1. Rarefaction curves of all barcoded *16s rRNA* amplicon samples at the genus level, using: (a) the archaeal primer set (ARC344F/Arch806R) and (b) the universal prokaryotic primer pair (Pro341F/Pro805R).

(a)

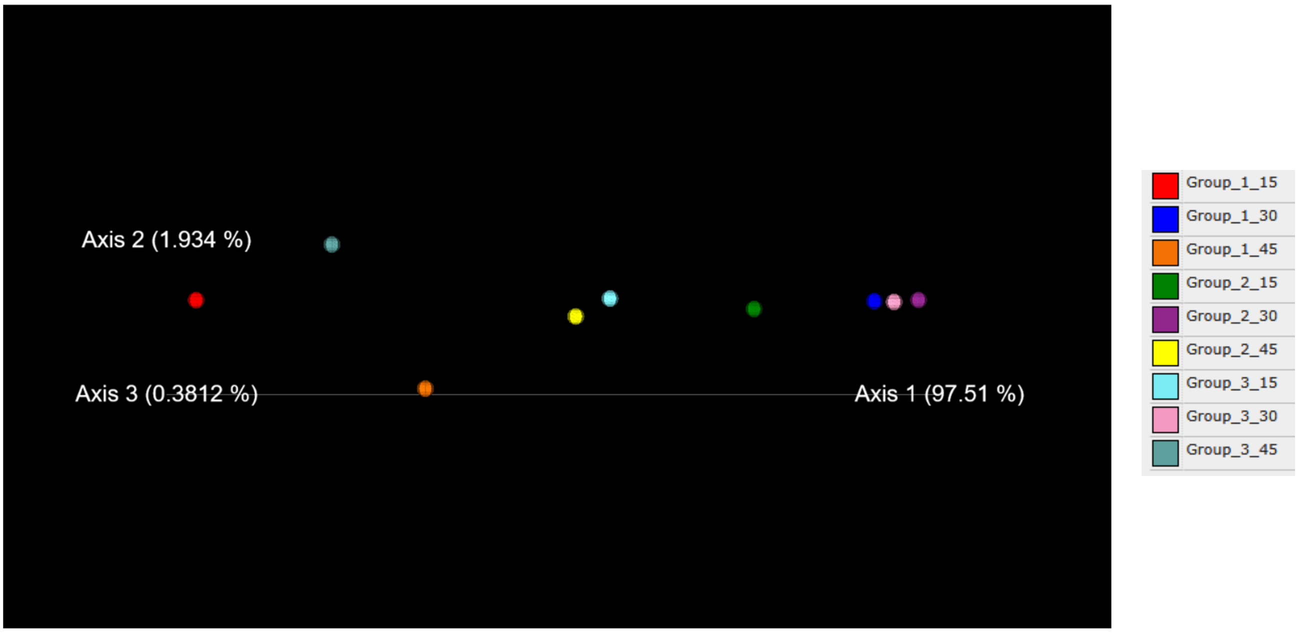

(b)

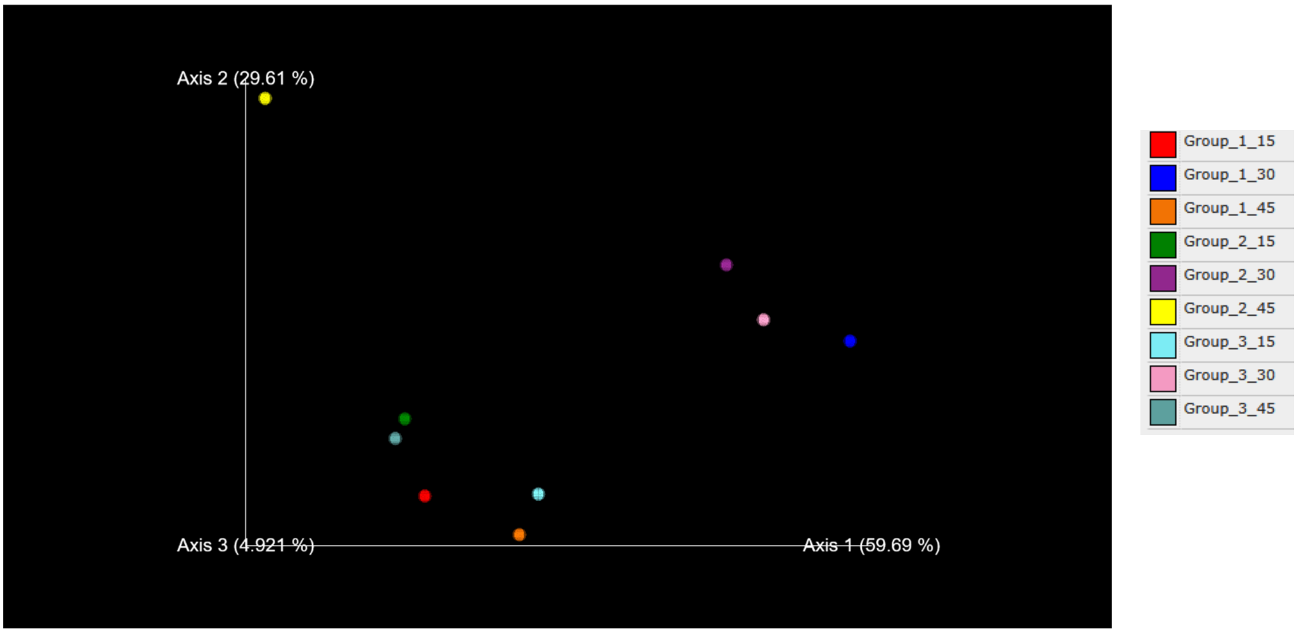

Figure S2. 3D Emperor plot for the nine sample groups under study, based on the Bray–Curtis dissimilarity index at the genus level, using: (a) the archaeal primer set (ARC344F/Arch806R) and (b) the universal prokaryotic primer pair (Pro341F/Pro805R).

Table S1. Shannon diversity index and Simpson's diversity index for the nine sample groups under study, using the archaeal primer set (ARC344F/Arch806R) and the universal prokaryotic primer pair (Pro341F/Pro805R).

Archaeal primer set (ARC344F/Arch806R)										
	**Sample groups**									
**Indices**	Group 1_15	Group 2_15	Group 3_15	Group 1_30	Group 2_30	Group 3_30	Group 1_45	Group 2_45	Group 3_45	Total
Shannon diversity index	1.63	1.49	1.38	1.21	1.29	1.48	1.58	1.45	1.66	1.51
Simpson's index	0.7	0.67	0.6	0.6	0.61	0.69	0.73	0.66	0.72	0.68
Prokaryotic primer pair (Pro341F/Pro805R)										
	**Sample groups**									
**Indices**	Group 1_15	Group 2_15	Group 3_15	Group 1_30	Group 2_30	Group 3_30	Group 1_45	Group 2_45	Group 3_45	Total
Shannon diversity index	3.09	3.1	2.87	2.77	3.23	3.05	2.94	3.33	3.12	3.17
Simpson's index	0.81	0.85	0.82	0.83	0.88	0.85	0.82	0.89	0.84	0.85
